# Differential Effects of Estrogen Receptor Alpha and Beta on Endogenous Ligands of Peroxisome Proliferator-Activated Receptor Gamma in Papillary Thyroid Cancer

**DOI:** 10.3389/fendo.2021.708248

**Published:** 2021-09-07

**Authors:** Shucai Yang, Zhongqin Gong, Zhimin Liu, Minghui Wei, Lingbin Xue, Alexander C. Vlantis, Yang Zhang, Jason YK. Chan, C Andrew van Hasselt, Xianhai Zeng, Shuqi Qiu, Nelson Tang, Jing Du, Wei Wei, Michael CF Tong, George G. Chen

**Affiliations:** ^1^Department of Clinical Laboratory, Pingshan District People’s Hospital of Shenzhen, Shenzhen, China; ^2^Department of Otorhinolaryngology, Head and Neck Surgery, The Chinese University of Hong Kong, Prince of Wales Hospital, Shatin, Hong Kong SAR, China; ^3^Department of Biochemistry and Molecular Biology, Faculty of Basic Medical Sciences, Chongqing Medical University, Chongqing, China; ^4^Department of Head & Neck Surgery, Cancer Hospital Chinese Academy of Medical Sciences, Shenzhen Center, Shenzhen, China; ^5^Shenzhen Key Laboratory of Ear, Nose and Throat (ENT), Institute of ENT & Longgang ENT Hospital, Shenzhen, China; ^6^Department of Chemical Pathology, The Chinese University of Hong Kong, Prince of Wales Hospital, Shatin, Hong Kong SAR, China; ^7^Department of Laboratory Medicine, Peking University Shenzhen Hospital, Shenzhen, China; ^8^Department of Thyroid and Breast Surgery, Peking University Shenzhen Hospital, Shenzhen, China

**Keywords:** papillary thyroid cancer, peroxisome proliferator-activated receptor gamma, estrogen receptors, PGJ2, 15(S)-HETE

## Abstract

**Purpose:**

The inhibition of estrogen receptor alpha (ERα) or the activation of ERβ can inhibit papillary thyroid cancer (PTC), but the precise mechanism is not known. We aimed to explore the role of ERα and ERβ on the production of endogenous peroxisome proliferator-activated receptor gamma (PPARγ) ligands in PTC.

**Methods:**

2 PTC cell lines, 32 pairs of PTC tissues and matched normal thyroid tissues were used in this study. The levels of endogenous PPARγ ligands 15(S)-hydroxyeicosatetraenoic acid (15(S)-HETE), 13-S-hydroxyoctadecadienoic acid (13(S)-HODE), and15-deoxy-Δ12,14-prostaglandin J2 (PGJ2) were measured by ELISA.

**Results:**

The levels of PGJ2 and 15(S)-HETE were significantly reduced in PTC, but 13(S)-HODE was not changed. Activation of ERα or inhibition of ERβ significantly downregulated the production of PGJ2, 15(S)-HETE and 13(S)-HODE, whereas inhibition of ERα or activation of ERβ markedly upregulated the production of these three ligands. Application of endogenous PPARγ ligands inhibited growth, induced apoptosis of cancer cells, and promoted the efficacy of chemotherapy.

**Conclusion:**

The levels of endogenous PPARγ ligands PGJ2 and 15(S)-HETE are significantly decreased in PTC. The inhibition of ERα or activation of ERβ can inhibit PTC by stimulating the production of endogenous PPARγ ligands to induce apoptosis in cancer cells.

## Introduction

There is increasing evidence indicating that activation of peroxisome proliferator-activated receptor gamma (PPARγ) by its ligands can inhibit the growth of thyroid cancer, likely *via* multi-mechanisms including stimulation of the anti-tumor immune system, induction of cancer cell differentiation, increase of radioiodine uptake in thyroid cancer cells, cell cycle arrest, and promotion of apoptosis of cancer cells (1–12). However, the rationale for administration of PPARγ ligands to treat thyroid cancer is not clear as some studies have shown a reduction in PPARγ expression, yet others revealed normal PPARγ expression or the occurrence of PAX8-PPARγ which can inactivate rather than decreasing PPARγ in thyroid cancer (13–19).

Therefore, the defect in PPARγ pathway needs further investigation. Moreover, some publications have also challenged the safety of synthetic PPARγ ligands that are currently employed as anti-tumor agents in most studies. The administration of synthetic PPARγ ligands is now known to produce some significant side-effects including an increased risk of bladder cancer and cardiovascular diseases (3, 20, 21). These adverse effects have limited the therapeutic application of synthetic PPARγ ligands.

It is known that estrogen receptors (ERs) are involved in the development of thyroid cancer that is predominant in females. Estrogen executes its functions usually through its traditional receptors (ERα and ERβ). The activation of either ERα or ERβ appears to be associated with different outcomes (22). In cancers, ERα is positively associated with cell proliferation/growth. In contrast, ERβ negatively regulates cell growth. Tumors develop in ERβ-knockout mice but not in wild type mice (23). Although both normal and malignant thyroid tissues are known to express ERα and ERβ, the level of ERα appears to be more pronounced in malignant thyroid tissues and the ratio of ERβ to ERα is significantly higher in normal thyroid tissues when compared to malignant thyroid tissues (24–31). The increased level of ERα has been shown to stimulate the growth of thyroid tumor cells whereas the increased level of ERβ can suppress their growth (27–31).

Although both ERs and PPARγ belong to the family of nuclear receptor proteins and both can regulate thyrocyte proliferation and growth, there are very few studies on the relationship between ERs and PPARγ in cancer cells. This study therefore aimed to examine the impact of ERs on endogenous PPARγ ligands in papillary thyroid cancer (PTC), the most common form of thyroid cancers. Endogenous PPARγ ligands are *in vivo* metabolic products which are nontoxic at physiological concentrations. Unfortunately, studies have not been actively conducted to explore the therapeutic modulation of these natural endogenous ligands for possible treatment of cancers.

## Methods

### Reagents

15(S)-hydroxyeicosatetraenoic acid (15(S)-HETE), 13-S-hydroxyoctadecadienoic acid (13(S)-HODE), 15-deoxy-Δ12,14-prostaglandin J2 (PGJ2), PGJ2 ELIS kits and 15(S)-HETE ELISA kits were purchased from Cayman Chemical (Ann Arbor, MI). 13(S)-HODE ELISA kits were from Enzo Life Sciences (Farmingdale, NY). 4,4’,4”-(4-propyl-[1H]-pyrazole-1,3,5-triyl) trisphenol (PPT, ERα agonist), 2,3-bis(4-hydroxy-phenyl)-propionitrile (DPN, ERβ agonist), 1,3-bis(4-hydroxyphenyl)-4-methyl-5-[4-(2-piperidinylethoxy)phenol]-1H-pyrazole dihydrochloride (MPP, ERα antagonist), 4-[2-phenyl-5,7-bis(trifluoromethyl)pyrazolo[1,5-a]pyrimidin-3-yl]phenol (PHTPP, ERβ antagonist) and paclitaxel were obtained from Tocris (Bristol, UK).

### Thyroid Tissue Samples

Papillary thyroid cancer (PTC) tissue samples of both tumor and non-tumor tissue from the same thyroid gland were collected from 32 patients including 6 males (35-58 years old) and 26 females (33-57 years old). All patients underwent routine thyroidectomy. All subjects provided written informed consent prior to specimen collection. Human Ethics approval (No. 2019.587) was obtained from the Joint Chinese University of Hong Kong-New Territories East Cluster Clinical Research Ethics Committee, and the study was performed in accordance with the 1964 Declaration of Helsinki.

### Cell Cultures

Two human PTC cells (K1 and BCPAP) were used in this study. K1 cells were obtained from the European Collection of Authenticated Cell Cultures (ECACC) and BCPAP cells were kindly provided by Dr. Mingzhao Xing (Johns Hopkins University School of Medicine, Maryland). Both cell lines have been authenticated to be human papillary thyroid cancer cells (32). K1 and BCPAP cells were cultured in RPMI 1640 supplemented with 10% FBS at 37° in an atmosphere with 5% CO2 and were used for the experiments in their early passages (less than 25). In our early study, we have demonstrated that both K1 and BCPAP cells can express certain basic levels of ERα, ERβ and PPARγ proteins (33).

### Cell Growth

The growth of cells was estimated by cell survival assay, which was determined by 3-(4,5-Dimethylthiazol-2-yl)-2,5-diphenyltetrazolium (MTT) protocol (33, 34).

### Measurement of PGJ2, 15(S)-HETE and 13(S)-HODE

The levels of PGJ2, 15(S)-HETE and 13(S)-HODE were determined by ELISA kits and the assays were performed according to the instructions of the manufacturers. Briefly, for tissue samples, they were measured wet weight and then homogenized in 2 ml 1 x PBS (pH 7.4) using a homogenizer on ice. For cultured cell samples, cells were lysed by lysis buffer (10 mM Tris-HCl, pH 7.4, 400 mM NaCl, 1 mM EDTA and 1.0% SDS) and samples were centrifuged at 5000 rpm for 1 min at 4°C to obtain the supernatant. The tissue homogenates or the lysed cell samples were acidified by adding 2M HCl to pH 3.5, left at 4°C for 15 min. Samples were centrifuged at 2000 rpm for 20 min at 4°C. Samples were applied to these C18 reverse phase column and the columns were washed with 10 mL water followed by 10 mL 15% ethanol, and 10 mL hexane. The sample was eluted from column by addition of 10 ml ethyl acetate and then evaporated under a stream of nitrogen. 25 μl ethanol and 250 μl Assay Buffer were added to dry samples. A standard curve was generated by serial dilutions of the standard supplied in these kits. The levels of the ligands were calculated according to the standard curve. Concentrations of 15(S)-HETE, 13(S)-HODE and PGJ2 were calculated by 4 parameter logistic curve fitting program.

### Analysis of Apoptosis

Cells were seeded in 6-well plates and incubated overnight to allow cells to attach to the plate. Terminal deoxynucleotidyl transferase Dutp nick end labeling (TUNEL) was conducted using an APO-DIRECT TUNEL assay kit (BD Biosciences, San Jose, CA). In brief, cells were suspended in 1% (w/v) paraformaldehyde in PBS, Ph7.4 at a concentration of 2×10^6^ cells/ml after treatment. The cell suspension was then placed on ice for 60 min. After centrifuging cells for 5 min at 300 g, the supernatant was discarded. The cells were washed in 5 ml of PBS and the cell pellet was resuspended in PBS in a tube by gentle vortexing. The cells were then incubated in ice-cold 70% (v/v) ethanol overnight at -20°C prior to staining for apoptosis. Apoptosis was measured according to the protocol provided by the kit and the result was presented as folds of control conditions.

### Statistical Analysis

Data were analyzed by student’s t test or one-way ANOVA followed by the student’s t test. All data were presented as means ± SD. The value was considered significant when p<0.05.

## Results

### The Levels of PGJ2, 15(S)-HETE and 13(S)-HODE in PTC

**Figure 1:**
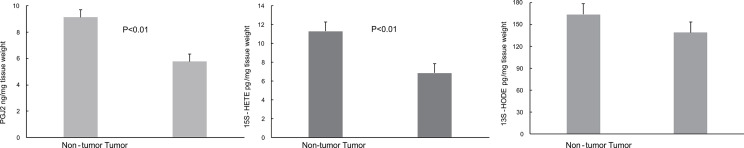
The concentrations of PGJ2 and 15(S)-HETE in PTC. Thyroid tumor tissues and its matched non-tumor tissues were obtained from 32 patients. The concentrations of PGJ2 and 15(S)-HETE were measured using ELISA kits from Cayman Chemical (Ann Arbor, MI) and Enzo Life Sciences (Farmingdale, NY). The ELISA was performed according to the instructions of the manufacturers. The concentrations of these 2 ligands were expressed in ng or pg per mg wet weight of tissues.

The concentrations of PGJ2 and 15(S)-HETE were much lower in PTC tumor tissues than in the non-tumor tissues (**Figure 1**). The concentration of 13(S)-HODE was decreased in PTC tumor tissues compared with that in the non-tumor tissues but the difference did not reach a significant point (p>0.05, data not shown).

### Impact of ER Modulation on the Levels of PGJ2, 15(S)-HETE and 13(S)-HODE in PTC Cells

**Figure 2:**
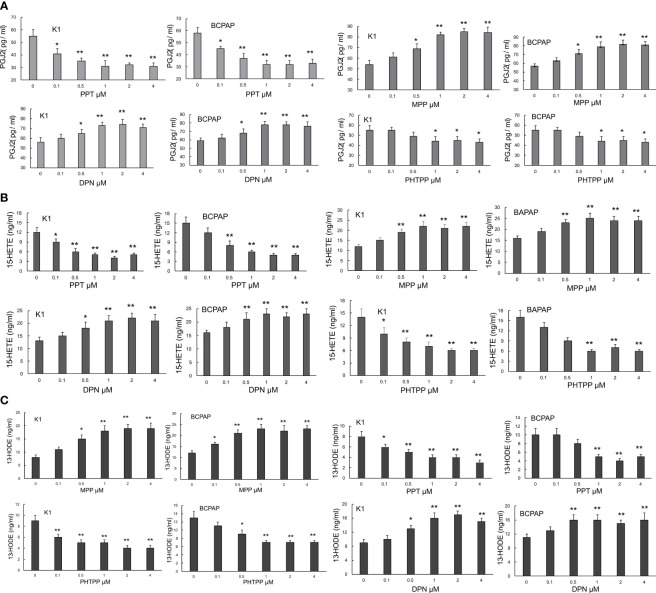
The impact of ER modulation on the levels of PGJ2, 15(S)-HETE and 13(S)-HODE in PTC cells. K1 and BCPAP cells were respectively treated with PPT, MPP, DPN and PHTPP at the given concentrations (0, 0.1, 0.5, 1, 2, 4µM for all 4 modulators) for 48 hours. At the end of the treatment, the levels of PGJ2 **(A)**, 15(S)-HETE **(B)** and 13(S)-HODE **(C)** in cells were measured by ELISA kits (Cayman Chemical (Ann Arbor, MI, and Enzo Life Sciences, Farmingdale, NY). The ELISA was performed according to the instructions of the manufacturers. *p < 0.05, **p < 0.01, compared with the control (0 dose).

In order to assess whether the production of endogenous PPARγ ligands, PGJ2, 15(S)-HETE and 13(S)-HODE, could be regulated by ERα and ERβ, PPT (ERα agonist), DPN (ERβ agonist), MPP (ERα antagonist) and PHTPP (ERβ antagonist) were employed in this study. These 4 agents have been well documented to modulate the activities of ERα and ERβ (35, 36). It was found that the activation of ERα by PPT markedly and dose-dependently inhibited the production of PGJ2 in both K1 and BCPAP cells. In contrast, the inactivation of ERα by MPP significantly and dose-dependently enhanced its production in both PTC cells (**Figure 2A**). Different from ERα activation, the activation of ERβ (by DPN) clearly increased the level of PGJ2 whereas the inactivation of ERβ by PHTPP decreased the PGJ2 production in both PTC cells (**Figures 2B, C**). Similar to PGJ2, the levels of 15(S)-HETE and 13(S)-HODE were regulated by these 4 ER modulators in both PTC cells. It appeared that their impact on 15(S)-HETE was more obvious than that on 13(S)-HODE.

### Impacts of the ER Modulation and Endogenous PPARγ Ligands on Cell Survival and Growth

**Figure 3:**
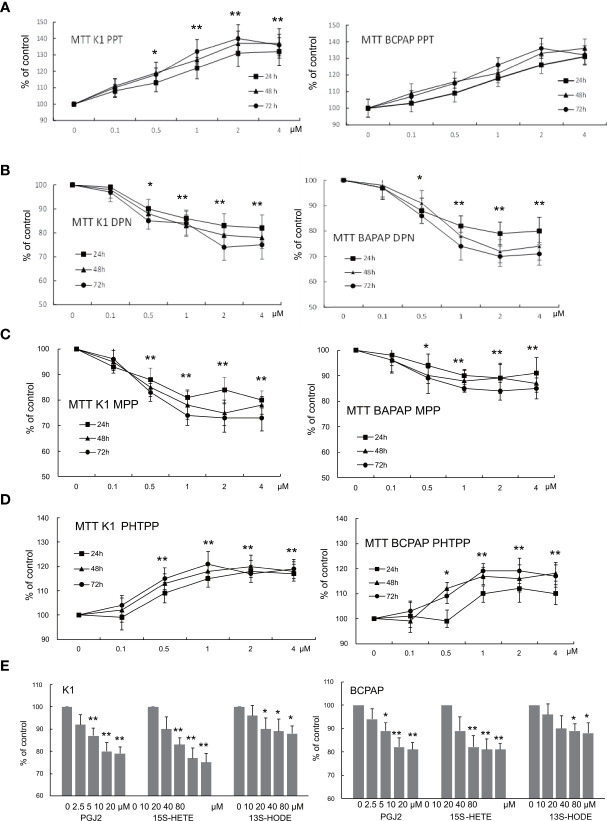
The impact of ER modulation and endogenous PPARγ ligands on cell growth. K1 and BCPAP cells were respectively treated with PPT **(A)**, DPN **(B)**, MPP **(C)**, and PHTPP **(D)** at the given concentrations for 24, 48 and 72 hours. At the end of the treatment, cell survival was measured by MTT assay to estimate the cell growth and expressed as the percentage of control culture conditions (no treatment). To assess the effect of endogenous PPARγ ligands on cell growth, different doses of PGJ2, 15(S)-HETE and 13(S)-HODE, as indicated in the figure, were used to treat K1 and BCPAP for 48 hours **(E)**, and cell growth was determined by the survival assay as described above. The data were presented as the mean ± SD of 3 independent experiments with triplicate wells. *p < 0.05, **p < 0.01, compared with the control (0 dose).

The modulation of ERα and ERβ exerted opposite effects on PTC cell survival and growth (**Figure 3**). The activation of ERα (by PPT) or inactivation of ERβ (by PHTPP) dose-dependently increased the survival and growth in both K1 and BCPAP cells (**Figures 3A, D**) whereas activation of ERβ (by DPN) or inactivation of ERα (by MPP) significantly decreased the survival and growth in both K1 and BCPAP cells (**Figures 3B, C**). All three endogenous PPARγ ligands markedly reduced the survival and growth in both PTC cells and the effects of PGJ2 and 15(S)-HETE were stronger than those of 13(S)-HODE (**Figure 3E**).

### Impact of ER Modulation and Endogenous PPARγ Ligands on Apoptosis

**Figure 4:**
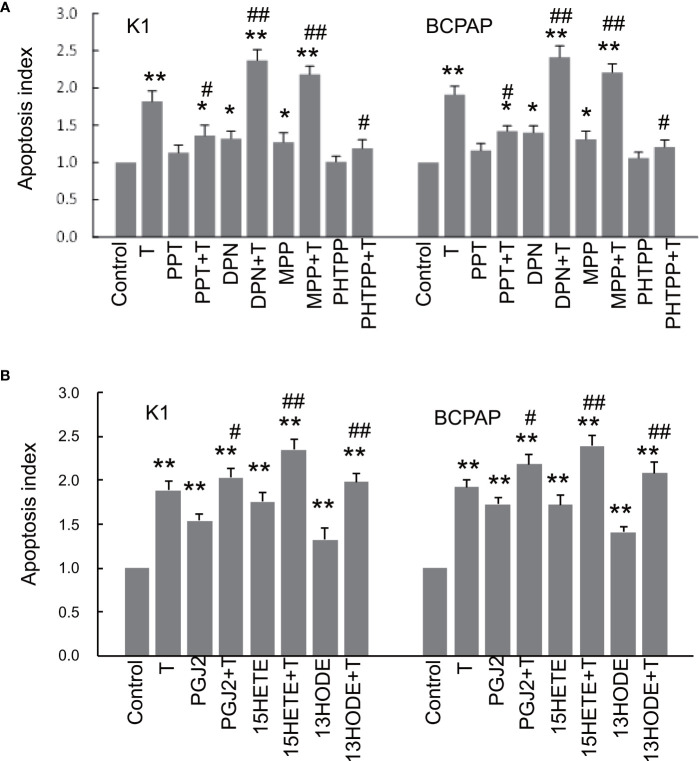
The impact of ER modulation and endogenous PPARγ ligands on apoptosis. K1 and BCPAP cells were respectively treated with 4 different ER modulators (1 µM PPT, 1 µM DPN, 1 µM MPP, 1 µM PHTPP), 1 µM paclitaxel (T) or ER modulator plus T for 48 hours **(A)**. At the end of the treatment, apoptosis was measured by TUNEL assay kits (BD Biosciences, San Jose, CA). The apoptotic index was calculated as folds of the control condition (no treatment). To assess the effect of endogenous PPARγ ligands on apoptosis, cells were respectively treated paclitaxel (T) or PPARγ ligand plus T for 48 hours **(B)**, and apoptosis was measured as described above. The data were presented as the mean ± SD of 3 independent experiments with triplicate wells. *p < 0.05, **p < 0.01 compared with the control (0 dose); #p < 0.05, ##p < 0.01 compared with cells treated with T only.

PTC cells treated with the ERα agonist PPT or ERβ antagonist PHTPP barely affected the apoptosis compared with those without PPT or PHTPP treatment (control) (**Figure 4A**). However, both PPT and PHTPP significantly sensitized the cells to apoptosis induced by paclitaxel, a chemotherapeutic agent that is commonly used in the treatment of thyroid cancer (37). The activation of ERβ (by DPN) or inactivation of ERα (by MPP) significantly stimulated apoptosis of PTC cells compared with the control, and both DPN and MPP further enhanced apoptosis induced by paclitaxel (**Figure 4A**). All three endogenous PPARγ ligands clearly induced apoptosis in both PTC cells and the effects of PGJ2 and 15(S)-HETE appeared to be stronger than that of 13(S)-HODE (**Figure 4B**). These three endogenous PPARγ ligands, especially 15(S)-HETE, also significantly enhanced the apoptosis induced by paclitaxel.

## Discussion

The results of this study have led to two novel findings. Firstly, the concentrations of endogenous PPARγ ligands, PGJ2 and 15(S)-HETE (38–41), were significantly reduced in PTC, though the level of 13(S)-HODE was not different between tumor tissues and non-tumor tissues. Secondly, the activation of ERα negatively controlled the production of endogenous PPARγ ligands whereas the activation of ERβ positively regulated them. These two novel findings are significant in elucidating the roles of PPARγ and ERs in the growth and potential treatment of PTC.

The activation of PPARγ ligands is well known to cause the death of cancer cells *via* multiple channels such as activating the anti-tumor immune system, differentiating cancer cells, arresting cell cycle, promoting apoptosis and increasing radioiodine uptake (1–12). The rationale for the application PPARγ ligands to treat thyroid cancer is inconsistent or unclear. Some studies have indicated that the expression of PPARγ is reduced in thyroid cancer while others revealed the normal expression of PPARγ or the inactivation of PPARγ by the Pax-8-PPAR-γ fusion protein (PPFP) (14–19, 33). If the low expression of PPARγ is the major factor that causes the PPARγ system unable to function normally, the proper treatment strategy should be to enhance the expression of PPARγ rather than the administration of PPARγ ligands. Thus, in such a situation, the administration of PPARγ ligands may not be an effective strategy to upregulate PPARγ functions. However, in practice, the induction of death in cancer cells is usually caused by the application of PPARγ ligands rather than by the upregulation of PPARγ itself. Therefore, the low expression of PPARγ itself is unlikely to be a key issue associated with the application of PPARγ ligands to treat thyroid cancer. Our study has demonstrated the decrease of endogenous PPARγ ligands, PGJ2, 15(S)-HETE and 13(S)-HODE in thyroid cancer. Insufficient PPARγ ligands can significantly downregulate the activity of PPARγ (38, 42), thus causing the PPARγ system unable to attack cancer cells. Accordingly, our findings have led to the discovery of a new pathway in which the activity of PPARγ is reduced by the low production of endogenous PPARγ ligands such as PGJ2, 15(S)-HETE and 13(S)-HODE. This new concept may well explain the rationale for the application of PPARγ ligands to treat thyroid cancer.

Earlier studies have demonstrated that the activation of ERα promotes the growth of PTC whereas the activation of ERβ inhibits the growth (27–30, 34). However, the responsible mechanism is not completely known. Our finding that the activation of ERα or inhibition of ERβ could significantly downregulate the production of endogenous PPARγ ligands, PGJ2, 15(S)-HETE and 13(S)-HODE, whereas inhibition of ERα or activation of ERβ could markedly upregulate the production of these three endogenous PPARγ ligands in PTC, uncovering new signaling pathways through which ERα and ERβ differentially regulate the levels of endogenous PPARγ ligands. Extensive studies have shown that the activation of PPARγ by its ligands (either synthetic or endogenous) can inhibit the growth of thyroid cancer (1–12, 40, 41). In this study, we have confirmed that the application of PPARγ ligands, PGJ2, 15(S)-HETE and 13(S)-HODE could inhibit the growth of PTC cells and promote apoptosis of tumor cells. Therefore, the inhibition of ERα or activation of ERβ may inhibit PTC by stimulating the production of endogenous PPARγ ligands to induce apoptosis in PTC cells. However, the upregulation of ERα or downregulation of ERβ may also promote the growth of PTC *via* decreasing the production of endogenous PPARγ ligands, which may also contribute to chemo-resistance. This novel concept is supported by a recent study which demonstrated that ERα signaling downregulates PPARγ to promote the progression of PTC (43). Nevertheless, we believe, this ER-regulated endogenous PPARγ ligand pathway should not be the sole pathway but one of channels for ERs to affect the growth of PTC or a certain subset of PTC.

The upregulation of endogenous PPARγ ligands such as PGJ2 and 15(S)-HETE appears to be a better strategy than the administration of a synthetic PPARγ ligand to inhibit thyroid cancer, at least in terms of side-effects. The administration of synthetic PPARγ ligands is associated with an increased risk of bladder cancer and other side effects (5, 20, 21). Endogenous PPARγ ligands are naturally produced *in vivo* and the cytotoxicity of these endogenous ligands should be minimal. Therefore, the development of endogenous ligands PGJ2 and 15(S)-HETE to treat thyroid cancer should be particularly appealing.

In conclusion, we have demonstrated that the levels of endogenous PPARγ ligands PGJ2 and 15(S)-HETE are significantly decreased in PTC. Our data suggest that the inhibition of ERα or activation of ERβ may inhibit PTC by stimulating the production of endogenous PPARγ ligands to induce apoptosis in cancer cells. Conversely, the upregulation of ERα or downregulation of ERβ may lead to the low production of endogenous PPARγ ligands, causing resistance of cancer cells to chemotherapy.

## Data Availability Statement

The original contributions presented in the study are included in the article/supplementary material. Further inquiries can be directed to the corresponding authors.

## Ethics Statement

Human Ethics approval was obtained from the Joint Chinese University of Hong Kong-New Territories East Cluster Clinical Research Ethics Committee. The patients/participants provided their written informed consent to participate in this study.

## Author Contributions

The experiments were designed by GC, SY, ZG, ZL, MT, and JC, and executed by SY, ZG, ZL, JD, and LX. The data analysis was conducted by GC, MT, SY, ZG, ZL, YZ, NT, JD, AV, CH, JC, and MW. Clinical samples and information were collected/provided by WW, AV, XZ, SQ, NT, and MW. The manuscript was written by SY, ZG, JC, ZL, MT, and GC with input from all of the other authors. All authors contributed to the article and approved the submitted version.

## Funding

This study was supported by grants from the National Natural Science Foundation of China (No.81972493), the Research Grants Council of the Hong Kong Special Administrative Region (Project No. GRF 14109716), the Project of Educational Commission of the Guangdong Province of China (Grant No: 2020KTSCX020) and the National Key R&D Program of China (Grant No:2020YFC2006400).

## Conflict of Interest

The authors declare that the research was conducted in the absence of any commercial or financial relationships that could be construed as a potential conflict of interest.

## Publisher’s Note

All claims expressed in this article are solely those of the authors and do not necessarily represent those of their affiliated organizations, or those of the publisher, the editors and the reviewers. Any product that may be evaluated in this article, or claim that may be made by its manufacturer, is not guaranteed or endorsed by the publisher.
